# Augmentation of Human Action Datasets with Suboptimal Warping and Representative Data Samples

**DOI:** 10.3390/s22082947

**Published:** 2022-04-12

**Authors:** Dawid Warchoł, Mariusz Oszust

**Affiliations:** Department of Computer and Control Engineering, Faculty of Electrical and Computer Engineering, Rzeszów University of Technology, W. Pola 2, 35-959 Rzeszów, Poland; marosz@kia.prz.edu.pl

**Keywords:** data augmentation, skeletal data, human action recognition, time series classification

## Abstract

The popularity of action recognition (AR) approaches and the need for improvement of their effectiveness require the generation of artificial samples addressing the nonlinearity of the time-space, scarcity of data points, or their variability. Therefore, in this paper, a novel approach to time series augmentation is proposed. The method improves the suboptimal warped time series generator algorithm (SPAWNER), introducing constraints based on identified AR-related problems with generated data points. Specifically, the proposed ARSPAWNER removes potential new time series that do not offer additional knowledge to the examples of a class or are created far from the occupied area. The constraints are based on statistics of time series of AR classes and their representative examples inferred with dynamic time warping barycentric averaging technique (DBA). The extensive experiments performed on eight AR datasets using three popular time series classifiers reveal the superiority of the introduced method over related approaches.

## 1. Introduction

The automatic interpretation of actions performed by the human body is both challenging and desired. Well-designed action recognition (AR) algorithms could be put into practice in the detection of aggressive behavior, video surveillance, interaction with humans and robots, or advanced control over virtual reality avatars. In recent years, many methods for human action recognition have been developed [1]. However, similarly to other subfields of pattern recognition, they suffer from overfitting or inability to create more robust machine learning models due to lack of diverse training samples. Therefore, the data augmentation techniques designed to enrich AR databases are desired. Furthermore, their usability in practice is also supported by the difficulty of creating AR databases with various samples covering feature space well enough to train a classifier. Consequently, the data augmentation methods used for multidimensional data samples (e.g., synthetic minority over-sampling technique (SMOTE) [2]) cannot be directly used for augmenting time series of AR classes since they take into account a relationship between consecutive measurements or often non-linear distortions affecting the duration variability of registered time series of a class [3]. Additionally, such time-related feature space prevents a simple addition of new data points (i.e., entire time series) between existing samples. Considering the challenges of the time series data augmentation techniques, in the literature, several approaches have been proposed. They perform operations that stretch, cut, shrink, or perturb input time series [4,5]. In more advanced solutions, new time-series are generated using deep network-based models [6], the weighting of aligned averages [7,8], or concatenating parts of two perturbed time series by the dynamic time warping (DTW) technique [9]. However, those methods are considering time series classification problems without addressing issues related to the AR time series domain, in which data samples often belong to a relatively large number of similar classes with irregular, partially-overlapping boundaries.

The literature review reveals the scarcity of time series augmentation approaches devoted to AR problems. Additionally, the existing solutions are often associated with mandatory data processing steps damaging important temporal information or architectures that require large-scale datasets and dedicated hardware for efficient training. Therefore, in this paper, a novel method for time series data augmentation is introduced. It uses SPAWNER (**S**ubo**P**tim**A**l **W**arped time series ge**NE**rato**R**) algorithm [9] to generate new data samples and incorporates a set of constraints to provide time series suitable for AR datasets. The constraints are defined to reject samples that do not introduce new knowledge to the dataset and samples likely to be generated in a solution space occupied by a neighboring class. To achieve such a goal, new time series is compared with one of its input samples and a representative solution created for a class using Dynamic Time Warping Barycenter Averaging (DBA) [7]. In the proposed **A**ction **R**ecognition SPAWNER (ARSPAWNER), the comparison is performed taking into account statistics of samples within a considered class.

The contributions of this study are as follows.

1. A novel method for AR time series augmentation with small amount of data;
2. A novel and efficient method for determining constraints on generated data samples using statistics for a class and its representatives along with their incorporation into the data augmentation approach to address AR-related characteristics;
3. Comparative evaluation of the method with related approaches on eight AR datasets using popular classifiers.

The paper is arranged as follows. Section 2 reviews previous work on human action recognition and time-series data augmentation. Section 3 introduces the proposed approach. In Section 4, feature extraction techniques used to process AR time series benchmarks employed in experiments are described. Section 5 presents comparative evaluation of the method with related approaches. Finally, Section 6 concludes the paper and indicates possible directions of future work.

## 2. Related Work

The classification results of a machine learning method depend on the availability of learning data samples. Hence, they should cover enough feature space to provide the classifier with information that allows for unequivocal determination of class labels of unknown samples. With only a few learning examples, the classifier in most cases would not be able to correctly infer differences between classes, identify class boundaries, or address the variability of samples within a class. Similarly, the imbalanced distribution of data samples per class or the occupation by the most samples of a small area may lead to a drop in the classification performance. Therefore, many approaches to enrich class diversity or determine artificial samples close to class boundaries are proposed based on linear data transformation [2,10]. However, such approaches cannot be used with time series as most of them are nonlinearly transformed in the time scale, which causes variations in their lengths, even for the same class. Hence, simpler approaches to time series augmentation consider removal of a part of a time series, adding data points between existing values (i.e., warping), or introducing noise, rotation, and scaling [4]. In a more developed solution proposed by Forestier et al. [8], DBA, averages of multiply aligned data samples are iteratively weighted. As a result, for a set of time series, a new example is generated that can be seen as their representative. However, its usage for time series of large dimensionality and length, aiming at generating more samples from selected subsets from the input dataset, is challenging due to its computation demands [7,9]. In the previous authors’ work, SPAWNER, time series are generated in the warped space between two data samples using their suboptimal alignment [9]. Specifically, the method uses DTW [11] for the alignment of perturbed parts of two input time series and concatenates aligned parts. The suboptimality arises from the usage of two randomly selected parts of each sample and the concatenation of their result instead of the DTW-based optimal alignment of the entire (i.e., non-divided) sequences. As those approaches are devoted to augmenting time series databases from many domains, there are works devoted to data generation techniques devoted to enriching time series from a single domain, addressing its characteristics. For example, Haradal et al. [6] introduced a method for augmentation of electrocardiogram (ECG) and electroencephalogram (EEG) datasets using generative adversarial networks (GAN) for the generation and discrimination of synthetic biosignals. In the work of Ramponi et al. [12], similar signals are generated with conditional GAN. The electrocardiograms are generated by Cao et al. [13] using samples of different classes and by Delaney et al. [14] using a variety of GAN architectures. Electroencephalographic data are augmented by Krall et al. [15] introducing distortions that consider temporal, spatial, or rotational changes. The data augmentation technique introduced by Le Guennec et al. [4] adds noise and magnitude changes to the input time series. Additionally, it warps them and removes some of their fragments (the cropping operation).

Some works address the augmentation of human action recognition datasets. For example, Shen et al. [16] proposed the Imaginative GAN (IGAN) and assessed it from a perspective of diversity and affinity of resulting samples. IGAN is a modification of the conventional GAN using unsupervised learning. The method approximates the distribution of the input data and samples new data. Additionally, it learns the latent behavioral (speed of actions) and physical (sizes of body parts) attributes. Ramachandra et al. [17] proposed an approach in which human activities measured by inertial sensors are recognized using data augmented by the proposed transformer GAN. Song et al. [18] specified an Interactive Action Translation (IAT) task that, taking into account rules of interaction, learns a model to generate a response for a given stimulation during inference. The method uses the Pair Embedding (PE) that utilizes Gaussian distributions of paired relationships to cluster individual actions in an embedding space and generate new pairs in their respective neighborhood. Here, encoders in a Paired Variational Auto-Encoders (PVAEs) and PCA-based linear dimension reductions are employed. Hoelzemann et al. [19] proposed human action data augmentation using a recurrent GAN based on a set of long short-term memory (LSTM) cells of four trained DeepConvLSTM models.

Despite promising performance of recent GAN-based data augmentation approaches, the GAN solutions require large-scale data to obtain stable models [16,18] or can be sensitive to outlying data samples [17]. Additionally, they may require data prepossessing in which human actions are unified to the same length due to architecture constraints. Consequently, the unification, or interpolation, negatively affects the input data and limits the variability of obtained samples. Furthermore, GAN, as other deep learning techniques, require demanding hyperparameter tuning [17], time-consuming training, and are associated with additional input data modifications to avoid overfitting.

Since, in this work, the augmentation of time series representing human actions is considered, main methods for their recognition are briefly introduced. They can be divided into deep learning and handcrafted approaches, where the techniques that belong to the first category extract suitable features and train a classifier in an end-to-end manner, while handcrafted approaches have separate feature extraction and classification steps. Furthermore, some of the deep learning methods are based on feature vectors but require a large amount of training data to provide acceptable models.

Among recently introduced AR methods, the approach by Sidor and Wysocki [20] uses a handcrafted Viewpoint Feature Histogram (VFH) point cloud description method [21] to calculate features for cells dividing point clouds of registered human actions. The cells represent different parts of the human body, and, therefore, such calculated features are more distinctive than those extracted for the whole cloud. Additionally, the method fuses two classifiers to improve its effectiveness. In the works of Pazhoumand-Dar et al. and Lillo et al. [22,23], the recognition is based on skeletal joint locations, angles between them, and more complex relationships between body parts. Skeletal data combined with local features extracted from depth images in the area around the projected joints can be found in the works of Raman and Maybank and Shahroudy et al. [24,25]. In these solutions, a two-level hierarchical Hidden Markov Model (HMM) [24] or hierarchical mixed norm with three levels of regularization over learning weights [25] are employed. One of the latest and most effective approaches to applying deep learning techniques to AR is presented by Farnoosh et al. [26]. In that work, a low-dimensional deep generative latent model encoding highly correlated skeletal data into a few sets of switching autoregressive temporal processes is introduced. The model decodes from the low-dimensional representations to the skeletal data and associated labels. Wang et al. [27] proposed the Skeleton Edge Motion Networks (SEMM) with spatio-temporal building blocks consisting of the concatenated spatial branch and temporal branch. It is observed that the spatial branch is effective when human actions do not have rich temporal information, while the temporal branch performs well with actions having a lot of movement of specific body parts. To boost the performance of SEMM, a progressive ranking loss that facilitates maintaining temporal order information in a self-supervised manner is employed. The spatial–temporal transformer network (ST-TR) is introduced by Plizzari et al. [28]. It models dependencies between skeletal joints using the transformer self-attention operator. Additionally, a spatial self-attention module (SSA) and a temporal self-attention module (TSA) are applied to understand intra-frame interactions between particular body parts and model inter-frame correlations. Then, the SSA and TSA are combined in a two-stream network. Donahue et al. [29] proposed an approach to human activity recognition based on video recordings using the long-term recurrent convolutional network (LRCN) with jointly trained convolutional (spatial) and recursive (temporal) parts.

In this study, to better highlight the capabilities of data augmentation techniques and offer results that can be easily replicated without additional hardware needed by recent deep learning models, handcrafted features, and popular classifiers are taken into account. Consequently, the relationship between generated samples of AR datasets that contain effective handcrafted features and the performance of several classifiers is investigated.

## 3. Proposed Method

In ARSPAWNER, two input time series of a given class are divided into two parts for a separate alignment using DTW and, after their concatenation, a new time series example is formed. This part of the time series processing is performed by the SPAWNER technique. Then, the resulted time series is rejected if it does not satisfy a set of constraints based on the AR time series characteristics.

In the approach, *M*-dimensional time series $X=[x^{1},x^{2},\ldots ,x^{L}]$ of the length *L* is processed. Specifically, each $x^{l}\in R^{M}$, l=1,2,…,L, and $X\in R^{L\times M}$. Then, a dataset of *N* samples, $L_{n},n=1,2,\ldots ,N$, $X_{n}\in R^{L_{n}\times M}$, $L_{n}$ is length of *n*-th sample, forms a collection $U=\{\left(X_{1},C_{1}\right),\left(X_{2},C_{2}\right),\ldots ,\left(X_{N},C_{N}\right)\}$, where C∈{1,K} are class labels (*K*). Consequently, a classifier trained on *U* assigns a label *C* to test time series $Y\in R^{L\times M}$.

To generate new time series based on a combination of two input samples $X_{1}$ and $X_{2}$ of the same class, the method employs DTW. In DTW, for $X_{1}=\left[x_{1}^{1},x_{1}^{2},\ldots ,x_{1}^{i},\ldots ,x_{1}^{L_{1}}\right]$ and $X_{2}=\left[x_{2}^{1},x_{2}^{2},\ldots ,x_{2}^{j},\ldots ,x_{2}^{L_{2}}\right]$, so-called *warping path* is determined which indicates optimal sequence $W=[w_{1},w_{2},\ldots ,w_{P}]$, where *P* is the length of the path, *p*-th element $w_{p}=\left(i,j\right)$, and $max\left(L_{1},L_{2}\right)\leq P<L_{1}+L_{2}$. Therefore, a $L_{1}\times L_{2}$ matrix *D* is calculated. For all (i,j), it contains distances between time series $\left[x_{1}^{1},\ldots ,x_{1}^{i}\right]$ and $\left[x_{2}^{1},\ldots ,x_{1}^{j}\right]$. To select the optimal alignment between $X_{1}$ and $X_{2}$, the path $W^{*}$ minimizing the total cumulative distance is found by calculating $D\left(i,j\right)={\left(x_{1}^{i}-x_{2}^{j}\right)}^{2}+min\left(D\left(i-1,j\right),D\left(i,j-1\right),D\left(i-1,j-1\right)\right)$. The warping path satisfies three conditions: (1) The boundary condition which forces the path to start at the beginning of the time series, $w_{1}=\left(1,1\right)$, and finish at their ends, $w_{P}=\left(L_{1},L_{2}\right)$; (2) The monotonicity condition according to which the time series indices in the path are monotonically increasing: ($i_{1}\leq i_{2}\leq \ldots \leq L_{1},j_{1}\leq j_{2}\leq \ldots \;\leq L_{2}$); (3) The continuity condition which limits the acceptable path steps to adjacent matrix elements. It can be written as $w_{p+1}-w_{p}\in \{\left(1,0,\left(0,1\right),\left(1,1\right)\right\}\forall_{p\in \{1,2,\ldots ,P-1\}}$. The warping window ξ limits the elements of $X_{1}$ and $X_{2}$ that can be aligned, i.e., $\forall_{\left(i,j\right)\in w_{p}}||i-j||\leq \xi$. DTW is used to calculate the distance $d=D(L_{1},L_{2})$ between time series.

To generate new examples in a suboptimal manner, an additional fourth constraint on the warping path is considered that forces it to contain the element $w_{p}=\left(R_{1},R_{2}\right)$, where $R_{1}=\left\lceil rL_{1}\right\rceil ,R_{2}=\left\lceil rL_{2}\right\rceil$, *r* is a single, uniformly distributed, randomly generated number in the interval (0,1). Here, ⌈·⌉ denotes ceiling operation. To prevent the calculation of $L_{1}\times L_{2}$ matrix *D* and reducing the computational cost, two matrices $R_{1}\times R_{2}$$D_{1}$ and $|\left(L_{1}-R_{1}\right)\left|\times \right|\left(L_{2}-R_{2}\right)|$$D_{2}$ are used. Then, $\left[x_{1}^{1},x_{1}^{2},\ldots ,x_{1}^{R_{1}}\right]$ is aligned with $\left[x_{2}^{1},x_{2}^{2},\ldots ,x_{2}^{R_{2}}\right]$ and $\left[x_{1}^{R_{1}+1},x_{1}^{R_{1}+2},\ldots ,x_{1}^{L_{1}}\right]$ is aligned with $\left[x_{2}^{R_{2}+1},x_{2}^{R_{2}+2},\ldots ,x_{2}^{L_{2}}\right]$. The resulting warping paths $W_{1}^{*}$ and $W_{2}^{*}$ are optimal due to the fourth constraint and the separate usage of $D_{1}$ and $D_{2}$. However, after their concatenation the obtained path is suboptimal. Moreover, $\xi_{1}$ and $\xi_{2}$ used to determine $W_{1}^{*}$ and $W_{2}^{*}$ are taken from $\left\lceil 0.1\cdot max\left(R_{1},R_{2}\right)\right\rceil$ and $\left\lceil 0.1\cdot max(\right|L_{1}-R_{1}\left|,\right|L_{2}-R_{2}\left|)\right\rceil$, respectively. They reduce the flexibility of the path from the perspective of the matrix *D*, as well as the concatenated paths $W_{1}^{*}$ and $W_{2}^{*}$. After the paths are concatenated to $W_{1,2}^{*}$, the algorithm aligns $X_{1}$ to $X_{2}$ generating sequences $X_{1}^{★}$ and $X_{2}^{★}$ with the length of $W_{1,2}^{*}$. To produce a new time series, $X^{★}$, $X_{1}^{★}$ and $X_{2}^{★}$ are merged, where $x^{★}\in X^{★}$, is a random number chosen from a normal distribution with a small σ, $x^{★}∼N\left(\mu ,\;\sigma^{2}\right)$, $\mu =0.5\left(x_{1}^{★}+x_{2}^{★}\right),\sigma =0.05\left|x_{1}^{★}-x_{2}^{★}\right|$.

To improve the quality of a AR dataset involving artificial example, $X^{★,C}$, generated by the method, additional constraints limiting the possibility of its acceptance are introduced. At first, average ${\tilde{d}}_{k}$ and standard deviation ${\hat{d}}_{k}$ of the DTW distances is computed between all samples that belong to each *k*-th (k=1,2,…,K) class. Then, the DBA approach is employed to provide representative sample for the class ${\overset{´}{X}}_{k}=DBA\left(X_{1}^{C},X_{2}^{C},\ldots ,X_{N}^{C}\right),C=k$, where $X_{N}^{C}$ is the number of samples that belong to the C=k class [8]. Specifically, it is computed as

(1) $$argmin{\overset{´}{X}}_{k}\in E\sum_{i=1}^{N^{C}}DTW^{2}\left({\overset{´}{X}}_{k},X_{i}^{C}\right),$$

where *E* is a space induced by DTW and the optimization problem is solved using an expectation-maximization scheme and iterative refining of the ${\overset{´}{X}}_{k}$ [8]. Finally, the $X^{★,C}$ is introduced to the dataset if the following conditions are met (Equations (2) and (3)):

(2) $$d_{1}>r_{1}{\tilde{d}}_{k}\wedge d_{2}>r_{1}{\tilde{d}}_{k},$$

(3) $$d_{1}<T\wedge d_{2}<T,$$

where $d_{1}=DTW\left(X_{1},X^{★,C}\right)$, $d_{2}=DTW\left({\overset{´}{X}}_{k},X^{★,C}\right)$, $T=r_{1}{\tilde{d}}_{k}+{\hat{d}}_{k}\left(r_{2}+{\hat{d}}_{k}/{\tilde{d}}_{k}\right)$, and $\left(r_{1},r_{2}\right)$ are parameters.

The proposed condition accepts only such time series which introduce new knowledge to the dataset, assuming that close proximity of the already present examples makes new examples redundant. The upper limit prevents the emergence of new examples in areas occupied by other classes.

**Figure 1:**
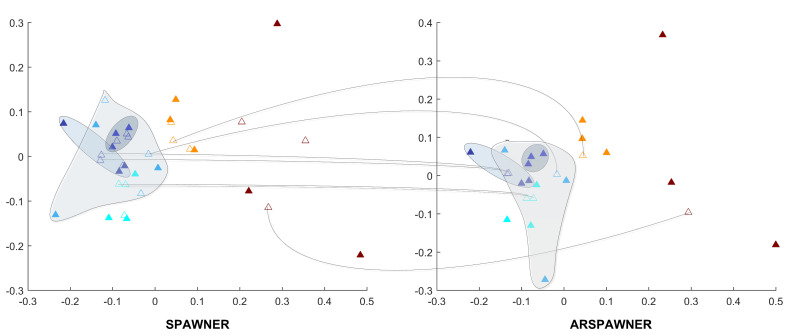
Class boundaries in the 2D MDS embeddings of DTW dissimilarities for the exemplary time series from the MSRA I dataset generated by SPAWNER and ARSPAWNER. Boundaries of neighboring classes are highlighted.

To highlight the differences between SPAWNER and ARSPAWNER, 2D Multi-Dimensional Scaling (MDS) [30] embeddings of DTW dissimilarities for the exemplary time series from the MSRA I dataset are presented in Figure 1. The figure contains class boundaries of similar or overlapping classes to better indicate areas in which the methods created new examples. Input data samples are denoted by filled triangles. To facilitate the analysis, the same examples are connected by arcs. As shown, the SPAWNER produces examples that are filtered out by ARSPAWNER. For example, two newly created members of the “orange” class by SPAWNER are rejected by ARSPAWNER due to their close proximity to the input data samples. Consequently, one member of the “green” class and three members of the “purple” class were rejected by ARSPAWNER. Interestingly, the scattered input examples of the “blue” class resulted in the emergence of two samples produced by SPAWNER that are too far from them. Hence, ARSPAWNER removed them, significantly altering the class boundary. It is worth noticing that the MDS embeddings strongly depend on the examples that are considered while it is calculated. Overall, the class boundaries with examples introduced by ARSPAWNER are compact, without time series that could negatively impact the recognition of samples from other classes.

## 4. Action Recognition Descriptors and Features

The action recognition features employed to show the effectiveness of the proposed data augmentation approach are using successful Bone Pair Descriptor (BPD) [31] and Distance Descriptor (DD) [32].

### 4.1. Distance Descriptor

The Distance Descriptor represents relationships among pairwise joint distances in skeletal data. DD can be calculated based on 3D joint coordinates, without using vector data. The descriptor features are obtained for *N* joints as follows.

1. For each joint $P_{i}$, 1≤i≤N do:
  (a) Calculate distances between the other joints $P_{j}$, j≠i;
  (b) Sort joints $P_{j}$ by the calculated distances in ascending order;
  (c) Assign consecutive integers $a_{ij}$ to the ordered joints $P_{j}$, starting from 1.
2. Create a feature vector consisting of integer values assigned to the joints $P_{j}$ in step 1(c) in the following order: $\left[a_{12},a_{13},a_{14},a_{15},a_{21},\ldots ,a_{NN-1}\right]$;
3. Reduce the feature vector by adding together integers *a* corresponding to the same pair of indices *i*, *j*: $\left[a_{12}+a_{21},a_{13}+a_{31},\ldots ,a_{N-1N}+a_{NN-1}\right]$.

Finally, each feature value is divided by 2(N−1) to normalize them to the interval [0–1]. Note that an input set of joints should be selected from the whole skeleton before the calculation of DD to reduce the computation time and increase the classification accuracy. DD is calculated using the Euclidean distance.

### 4.2. Bone Pair Descriptor

**Figure 2:**
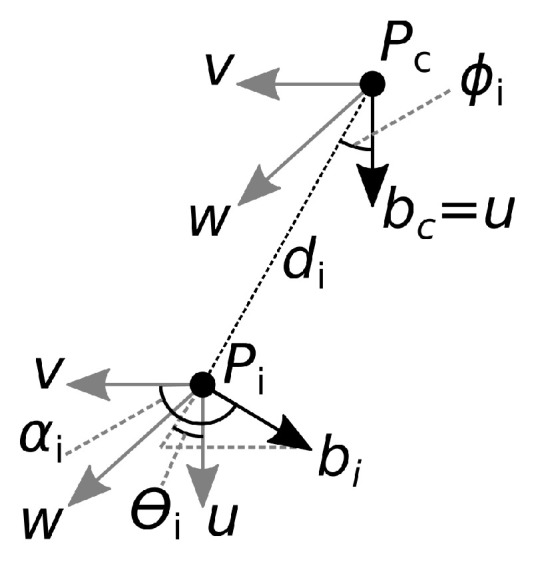
Calculation of Bone Pair Descriptor.

The Bone Pair Descriptor encodes the angular relations between particular pairs of bones. The descriptor is calculated as follows. Let $P_{c}$ be the skeleton central joint, $b_{c}$ the central vector associated with the joint $P_{c}$, $P_{i}$ the *i*-th non-central joint, and $b_{i}$ the vector associated with that joint (Figure 2). Vectors $b_{c}$ and $b_{i}$ coincide with a bone or a part of the spine.

The relative position of vectors $b_{c}$ and $b_{i}$ is described by α, ϕ, and Θ according to Equations (4)–(6) [33]:

(4) $$\alpha_{i}=a\cos\left(v_{i}\cdot b_{i}\right)$$

(5) $$\phi_{i}=a\cos\left(u\cdot \frac{d_{i}}{|d_{i}|}\right)$$

(6) $$\Theta_{i}=a\tan\left(\frac{w_{i}\cdot b_{i}}{u\cdot b_{i}}\right)$$

where the vectors *u*, $v_{i}$, and $w_{i}$ define the Darboux frame [34]:

(7) $$u=b_{c}$$

(8) $$v_{i}=\frac{d_{i}}{|d_{i}|}\times u$$

(9) $$w_{i}=u\times v_{i}$$

with · and × representing the scalar product and the vector product, respectively. Let *N* be the number of non-central joints. The BPD has 3N features calculated for each non-central joint using Equations (4)–(6):

(10) $$V=[\alpha_{1},\phi_{1},\Theta_{1},\alpha_{2},\phi_{2},\Theta_{2},\ldots ,\alpha_{N},\phi_{N},\Theta_{N}]$$

Finally, the features are normalized to the interval [0–1], dividing them by the maximum of π for α or ϕ, and 2π for Θ. BPD requires the selection of central joint $P_{c}$, non-central joints $P_{i}$, and joints determining vectors, $b_{c}$$b_{i}$, from the whole skeleton.

In the experiments, only α and ϕ features were used since Θ proved ineffective and its calculation is time-consuming [31].

## 5. Experiments and Discussion

### 5.1. Datasets

For the evaluation of the approach, six human action datasets with skeletal data were used: MSR Action3D (MSRA) [35], UTD Multimodal Human Action Dataset (UTD-MHAD) [36], UTKinect-Action3D (UTK) [37], Florence 3D Action Dataset (FLORENCE) [38], SYSU 3D Human–Object Interaction Set (SYSU) [39], and Kinect Activity Recognition Dataset (KARD) [40]. The MSRA dataset is split into three separate subsets, MSRA I, MSRA II, MSRA III, as suggested by its authors [35]. Each subset contains different action classes, although some of them appear in two subsets. That makes a total of eight datasets used in experiments. Detailed information about the datasets, including the length variability of time series, the number of input examples, and the number of augmented examples produced by each approach, is presented in Table 1.

According to the original paper introducing the MSRA dataset, there are seven subjects performing actions. However, the larger version, consisting of 10 subjects, is publicly available and can be downloaded from the authors’ website [41]. This version was used in the experiments.

In this study, two types of validation were performed. For MSRA, SYSU, UTD-MHAD, and KARD, 50-50 validation tests were used, in which the training and testing sets were split in half based on the subjects performing actions. The protocol for UTD-MHAD and FLORENCE is 10-fold cross-validation. For each dataset, the validation protocols proposed by the authors were used. In the case of KARD, 50-50 validation was used instead of the 10-fold cross-validation due to excessive computation time. All performed tests were subject independent, which means that in each test, the training set contains actions performed by subjects not present in the testing set. Such tests simulate the behavior of a recognition application in practice, where people performing actions do not participate in the creation of the training data.

**Figure 3:**
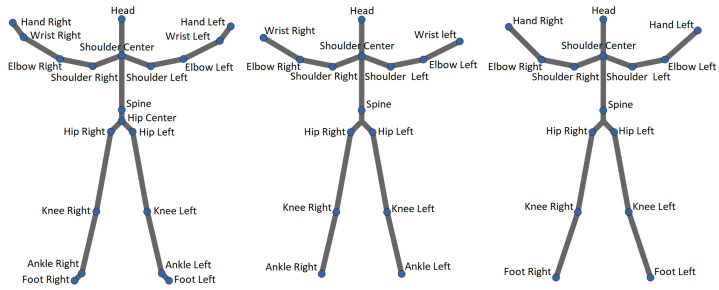
Three skeletons available in datasets: (**left**) MSRA, UTD-MHAD, UTK, and SYSU; (**middle**) FLORENCE; (**right**) KARD.

Actions from all datasets were recorded using a Microsoft Kinect sensor. In this work, only skeletal joints were used to characterize human actions. The skeletons for actions present in all datasets except FLORENCE and KARD consist of 20 joints, while the skeletons used to capture actions in the FLORENCE and KARD datasets consist of 15 joints (see Figure 3).

The same subsets of joints and bones cannot be used for 20-joint datasets and 15-joint datasets. Furthermore, FLORENCE and KARD do not have identical joint sets despite having the same number of joints. Therefore, for the experiments, three groups of joint subsets and bone subsets were selected separately for the Distance Descriptor and the Bone Pair Descriptor. They are listed in Table 2 and Table 3.

The subsets of joints and bones were selected experimentally as a part of the previous work on the subject of human action recognition [31]. Different configurations were also tested, however, the chosen subsets yielded the best results in terms of recognition rate and computation time.

### 5.2. Visual Examples of Augmented Time Series

**Figure 4:**
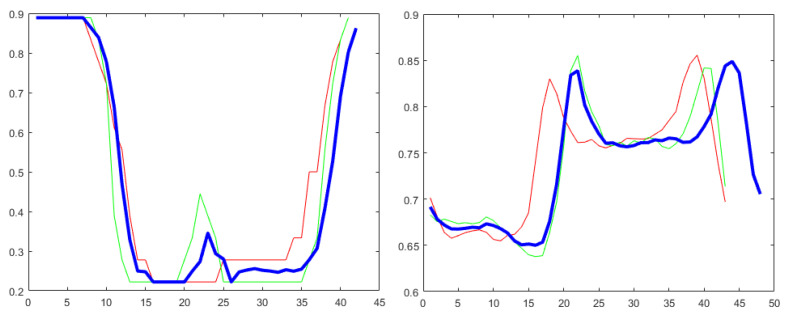
Time series generated by ARSPAWNER (blue curve) based on two exemplary timeseries (red and green curves). The left plot represents “draw circle” action and the right plot represents “high arm wave” action from MSRA II dataset.

To show exemplary time series, in Figure 4, two actions from the MSRA II dataset [35] (i.e., “draw circle” and “high arm wave”) are presented along with the additional time series generated by ARSPAWNER. The curves of the first action represent the first DD feature related to Hand Left and Hand Right joints, and the curves of “high arm wave” action represent ϕ feature of BPD, for which the non-central vector is determined by Wrist Right and Hand Right joints.

### 5.3. Classifiers

Among classification methods, the classical Dynamic Time Warping (DTW) and two recent methods were used: LogDet Divergence-based Metric Learning with Triplet constraints (LDMLT) [42] and Time series Cluster Kernel (TCK) [43]. LDMLT is a classifier based on Mahalanobis distance and the so-called triplet constraints used for its learning [42]. TCK is a method that calculates similarities between time series using Gaussian Mixture Models (GMM) augmented with informative prior distributions. It can handle missing data without the usage of imputation methods [43]. The output of DTW and LDMLT is the distance between two given sequences, i.e., each testing sequence is compared to each training sequence. Therefore, there is a need to apply the nearest neighbor classifier to determine the class represented by the closest sequence.

In Table 4, the configuration of parameters for each classifier is presented. The parameter values were set experimentally in the spirit of fairness, i.e., by changing them and checking whether the recognition rate is improved.

### 5.4. Results

The feature vectors used in the experiments are concatenations of the DD and BPD features without Θ, which makes a total of 69 features (45 for DD and 24 for BPD).

ARSPAWNER generates new data based on a pair of input time series, and therefore, the number of generated examples by other methods is aligned with the number of returned samples. This ensures a fair comparison of algorithms.

In this study, four augmentation methods are compared using the classification accuracy obtained for each dataset and classifier. Due to the randomness of the augmentation algorithms and TCK classifier, each accuracy is calculated for 10 runs and averaged. Then, the following values are calculated: average accuracy, average rank, geometric average rank, and a number determining how many times a method achieved the best accuracy (count best). These values are considered as the comparative criteria. To compare the methods, ranks from 1 to 5 are used, where a lower rank means a method has greater accuracy. The compared approaches are: SPAWNER, ARSPAWNER, Window Slicing [4], and Window Warping [4]. The results for each method, and for the case in which the augmentation is not performed (non-augmentation case), are presented in Table 5.

The experimental results reveal that the proposed ARSPAWNER is the most effective augmentation method and outperforms the non-augmentation case according to all comparative criteria. The method shows the greatest advantage over the others when used with DTW. However, in the case of the other two classifiers, ARSPAWNER and SPAWNER have close average effectiveness. They both significantly outperform the other methods, as well as the results of the non-augmentation case.

The LDMLT classifier yielded better results than the other two methods for all datasets, and its suitability for the action recognition problems was proven in the previous study [31]. The study of Kamycki et al. [9], in which SPAWNER was introduced, does not address action recognition problems considering time series from different domains. Interestingly, in that study, the LDMT classifier showed inferior performance. From Table 5, it can also be seen that the TCK classifier obtained the worst results among all three methods for all datasets, except UTK, for which it outperformed DTW.

Overall, it can be seen that the introduced ARSPAWNER outperforms the remaining data augmentation methods on action recognition datasets, since it considers the specificity of such data collections, with many similar and overlapping action classes.

### 5.5. Visual Comparison

**Figure 5:**
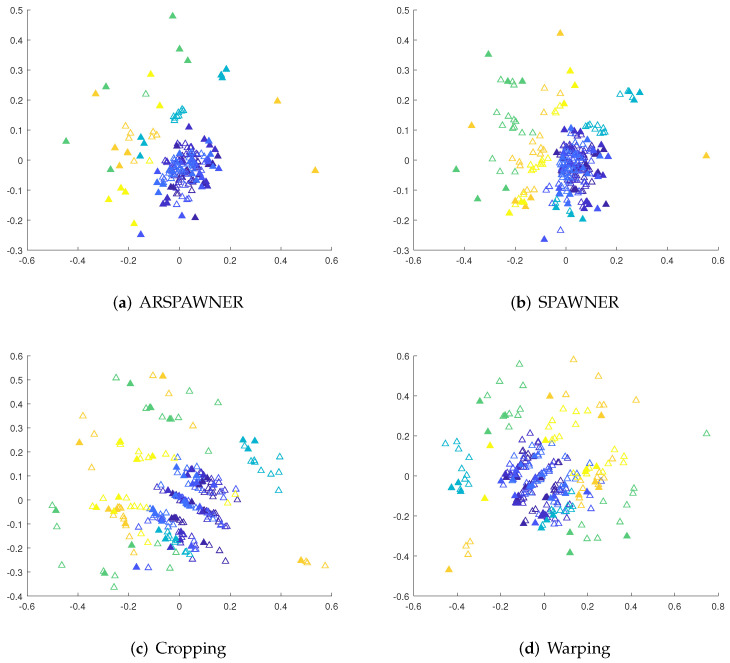
The 2D MDS embeddings of DTW dissimilarities between training and augmented sequences from the MSRA I dataset for the compared augmentation methods. Colors are used to differentiate the classes, the filled triangles denote input examples.

To show the areas in which new samples are generated by the augmentation methods from the MSRA I dataset, Kruskal’s nonmetric MDS [30] is employed. To facilitate the analysis, the first 60 actions are considered. MDS reduces the dimensionality of data samples and can be used with time series of different lengths by the usage of the DTW dissimilarity matrix. The matrix contains pairwise DTW distances between examples. The MDS representations of exemplary time series are shown in Figure 5. Input time series are filled while the colors indicate their classes. The proximity of samples from different classes or existing overlapped class boundaries illustrate the recognition problems. However, the introduction of new data samples in most cases improved the recognition accuracy of classifiers, it can be assumed that methods generating time series in areas within class boundaries are likely to lead to a higher recognition rate. As presented, ARSPAWNER generates fewer examples in areas occupied by representatives of other classes than in the case of the remaining augmentation approaches.

**Figure 6:**
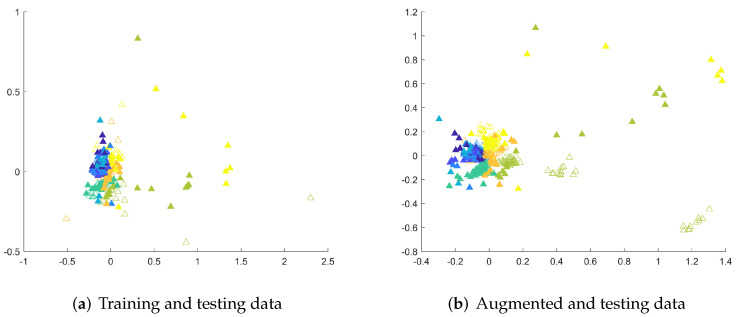
The 2D MDS embeddings of DTW dissimilarities between testing and training or testing and augmented sequences from the MSRA I dataset. Colors differentiate the classes, the filled triangles denote testing examples.

The recognition problems can also be highlighted by showing testing examples together with training data and augmented data. Therefore, in Figure 6, solid triangles represent 2D MDS embeddings of testing samples from the entire MSRA I dataset, and empty triangles denote training data (Figure 6a) and augmented data (Figure 6b), respectively. The placement of testing samples in the feature space indicates recognition problems as the class boundaries are difficult to determine due to the presence of clusters of similar examples from different classes in close proximity. Even classes that seem to be easily distinguished, represented here by yellow and bright green triangles are close to each other while training examples of the bright green class are far from that boundary (Figure 6a). This means that training examples do not carry enough information to be able to successfully recognize examples from these two classes. The emergence of augmented samples (Figure 6b) cannot solve this problem, since such knowledge cannot be obtained, but adds more examples in vital areas, shrinking overlapped class areas. Similar observations can be made for other datasets. It is worth noticing that the reported results strongly depend on the capabilities of used classifiers. Some of them may not be suitable to recognize human actions as can be seen in the TCK case.

### 5.6. Comparison with CGAN

Since there are approaches based on GAN architecture to augment time series in different domains, the performance of ARSPAWNER is compared with those of Conditional GAN on three MSRA datasets. Due to the lack of Matlab implementations of GAN-based approaches designed to augment action recognition time series in the literature, the available Matlab CGAN example designed to generate synthetic time series was adapted (MathWorks, https://www.mathworks.com (accessed on 13 March 2022)) [44]. The employed CGAN uses 1-D convolutional networks and is designed to perform the two-class augmentation. The generator network projects and reshapes the 1 × 1 × 100 noise arrays to 4 × 1 × 1024 arrays. It converts data labels to embedding 4× 1 × 1 vectors. Then, it concatenates the outputs of the two inputs and upsamples them to 1201 × 1 × 1 arrays with 1-D transposed convolution layers and ReLU layers. The dimensionality of the arrays is determined by the application of the origin of the adapted example. The discriminator network takes two inputs and classifies original and synthesized 1201 × 1 × 1 signals. It reshapes and concatenates them. Then, after downsampling, a series of 1-D convolution layers with leaky ReLU (a scale of 0.2) are employed.

The network was adapted to perform the augmentation of action recognition MSRA datasets that contain time series of different lengths, belonging to 8 classes and composed of 69 features. Specifically, due to the ability to generate two class time series, it was run eight times with input samples divided into two classes (i.e., the class considered in a given run and the rest). Additionally, since it is not designed to process multivariate time series and to avoid time-consuming computations, the PCA technique was applied to reduce the feature dimensionality from 69 to 5 and CGAN was run for each new feature independently with the concatenation of data to form synthesized five-dimensional vectors. Furthermore, since time series in MSRA datasets are of different lengths, they were interpolated to the same length, imposed by the network architecture. The finally obtained augmented examples were added to the original samples and employed by the nearest neighbor classifier with the DTW distance. The parameters of the network were set as recommended by the network designers, with the reduced number of iterations since the model converged earlier, allowing for the reduction in the training time. Important parameters of CGAN: number of iterations = 1000, learning rate = 0.0002, the Adam optimizer, batch size = 256, latent dimension = 100, and embedding dimension = 100. In experiments with CGAN, a PC with Nvidia Quadro RTX 4000 MAX-Q GPU, i9-10885H CPU, and 64 GB RAM was used. To ensure a fair comparison, ARSPAWNER was also run on the same five-dimensional feature vectors resulting from PCA.

**Figure 7:**
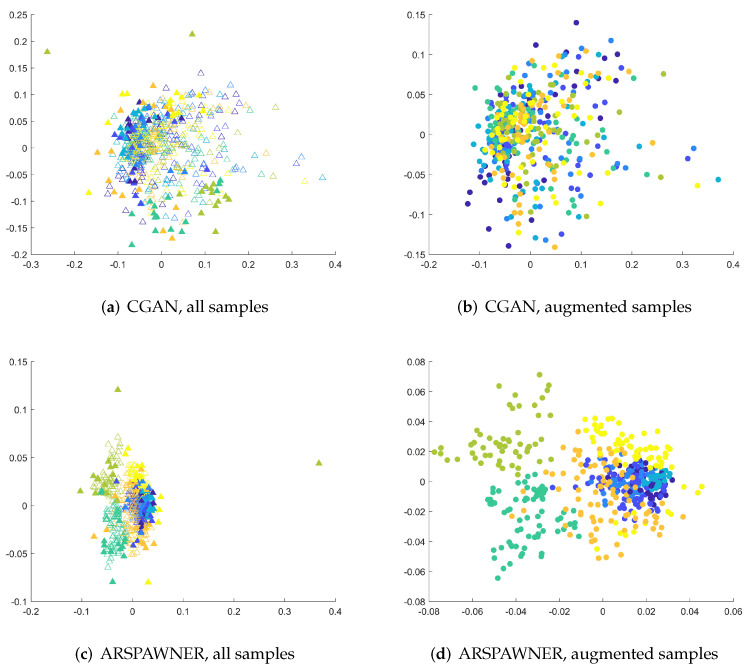
The 2D MDS embeddings of DTW dissimilarities between sequences of reduced dimensionality from the MSRA I dataset for CGAN and ARSPAWNER. Colors are used to differentiate the classes, the filled triangles denote input examples (**a**,**c**), while filled circles denote augmented samples (**b**,**d**).

The accuracy of the classifier for three augmented MSRA datasets after PCA feature reduction is presented in Table 6. It can be seen that the classifier equipped with data generated by ARSPAWNER improves its accuracy by a large margin. The improvement can also be visible for CGAN-created data in the case of MSRA I. However, for the MSRA II and III datasets, creating synthetic samples led to a significant drop in the performance of the classifier. The problems with the generation of suitable data examples of CGAN are possibly caused by the lack of a sufficient number of learning data examples, challenging data examples in the dataset after reduction by PCA, and inefficiency of the employed network architecture. To better highlight encountered problems with CGAN architecture, the 2D MDS embeddings were created for the entire MSRA I dataset (Figure 7). As shown, input data samples are close to each other due to the usage of PCA reducing the dimensionality of the time series in the dataset. However, ARSPAWNER was able to create samples in large clusters (Figure 7d) in their proximity (Figure 7c). CGAN, in turn, created many samples across the feature space, with their representatives also located in places that belong to the neighboring classes (Figure 7a,b).

### 5.7. Impact of Parameters

**Figure 8:**
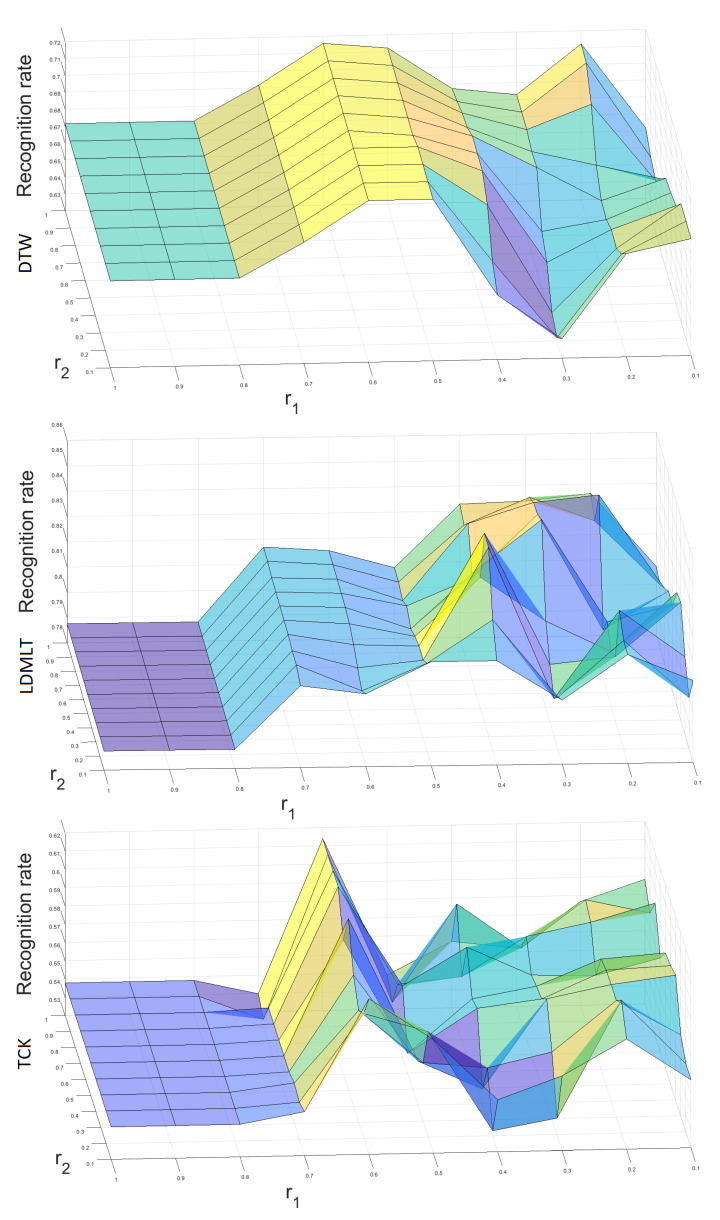
Three-dimensional surface plots presenting the impact of ARSPAWNER parameters $r_{1}$ and $r_{2}$ on classification accuracy with MSRA II dataset. The upper, middle, and lower plots represent the results of DTW, LDMLT, and TCK, respectively.

The next experiment concerns the impact of the ARSPAWNER parameters $r_{1}$ and $r_{2}$ on the classification accuracy. Figure 8 shows 3D surface plots calculated for each classifier and MSRA II dataset. The values of $r_{1}$ and $r_{2}$ are within the range [0.1–1.0] with step 0.1. The classification accuracy for DTW ranges from 63.2% to 72.6%, for LDMLT the range is [78.8–85.5%], and for TCK the range is [53.3–62.4%]. For each classifier, the difference between the lowest and the highest result is greater than 5 percentage points and smaller than 10 percentage points. Therefore, it can be concluded that the parameters $r_{1}$ and $r_{2}$ have a moderate impact on the classification accuracy.

The $r_{1}$ and $r_{2}$ parameters govern two constraints on the generated time series. Hence, a more detailed experiment, involving all three MSRA datasets, shows the impact of lower and upper constraints on the performance of ARSPAWNER with the nearest neighbor classifier with the DTW distance. Additionally, it allows for assessing the importance of the class representatives used in the conditions. The results presented in Table 7 indicate that both conditions should be present to obtain the best recognition rate for the MSRA datasets. However, the condition that rejects examples created near to a given input sample or a representative sample of a class (Equation (2)) is more influential than the upper limit (Equation (3)), responsible for acceptance of candidates closer to the class borders. Since both conditions are based on two distances to a considered input sample ($d_{1}$) and the DBA representative ($d_{2}$), their calculation reveals that they both should be used. It is justified by a larger drop in the performance of the ARSPAWNER in the case in which distance to the input sample is not employed. This confirms the usability of the introduced usage of the representative time series for each class.

### 5.8. Performance with Small Number of Training Examples

**Figure 9:**
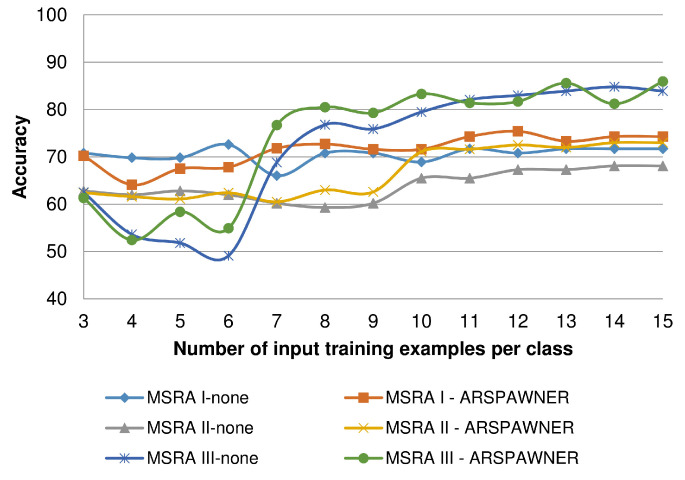
Average accuracy of the nearest neighbor classifier with the DTW distance based on a small number of augmented training examples per class.

To determine the capability of the introduced ARSPAWNER to augment small datasets, composed of a small number of training examples per class, it was tested using the MSRA I-III datasets varying the number of input time series. This experiment also indicates problems with small benchmark datasets in which class boundaries cannot be easily established due to an insufficient amount of available data and a relatively large number of classes (i.e., there are eight classes in the MSRA datasets). In the experiment, 3 to 15 input examples per class were randomly selected and used by ARSPAWNER to generate synthetic data. Then, the average accuracy of the nearest neighbor classifier with the DTW distance based on ten draws is reported in Figure 9. Overall, as reported, ARSPAWNER can improve the results of the classifier based only on a few available training samples. Depending on the dataset and the way testing examples are scattered in the feature space, the positive effect of the augmentation is visible even for five input examples.

## 6. Conclusions

In this paper, a novel method for the augmentation of datasets with time series representing human actions has been presented. The introduced ARSPAWNER improves the original SPAWNER by introducing action recognition-related constraints addressing problems present in this domain. The approach identifies data samples, i.e., time series, that are far enough from input samples and still do not cross the boundaries of other classes. Additionally, data samples that are in the proximity of the input time series, and consequently do not introduce new knowledge, are rejected. The constraints are based on distances between a new sample and an input sample and a sample generated as a representative time series characterizing a class. It has been shown that the introduced constraints provide to the augmentation leading to the improved performance of classifiers. The method has been experimentally compared with related approaches using three classifiers on eight action recognition datasets.

Future work will involve an application of optimization techniques to select a suitable set of generated time series based on data clustering quality indices. Such an approach can be seen as an extension of the study presented in this paper since constraints that remove augmented samples may be replaced with a step in which their suitability is assessed based on the quality criteria describing clusters of generated samples. Another interesting research direction is to employ augmentation methods like ARSPAWNER to augment small datasets and train time-consuming deep learning classifiers.

To facilitate the reproducibility of the approach, the Matlab implementation of the introduced ARSPAWNER is available at www.marosz.kia.prz.edu.pl/ARSPAWNER.html (accessed on 13 March 2022). The scripts for Distance Descriptor and Bone Pair Descriptor are also publicly available and can be downloaded [45].

## Figures and Tables

**Table 1:** Characteristics of datasets used in experiments.

Name	Classes	Subjects	Sequences (Actions)	Time Series Length	Input Examples	Augmented Examples	Validation Protocol
MSRA I	8	10	224	13–76	118	611	50-50 validation
MSRA II	8	10	207	15–100	118	573	50-50 validation
MSRA III	8	10	225	13–71	113	438	50-50 validation
UTD-MHAD	27	8	861	41–125	431	1163	50-50 validation
UTK	10	10	199	5–110	179	744	10-fold cross-validation
FLORENCE	9	10	215	8–35	194	1109	10-fold cross-validation
SYSU	12	40	480	58–638	240	1087	50-50 validation
KARD	18	10	540	42–310	270	685	50-50 validation

**Table 2:** Subsets of joints used for the Distance Descriptor. “L.” and “R.” denote Left and Right, respectively.

MSRA, UTD-MHAD, UTK, SYSU	FLORENCE	KARD
Hand L.	Wrist L.	Hand L.
Hand R.	Wrist R.	Hand R.
Shoulder L.	Shoulder L.	Shoulder L.
Shoulder R.	Shoulder R.	Shoulder R.
Head	Head	Head
Spine	Spine	Spine
Hip L.	Hip L.	Hip L.
Hip R.	Hip R.	Hip R.
Ankle L.	Ankle L.	Foot L.
Ankle R.	Ankle R.	Foot R.

**Table 3:** Subsets of bones used for the Bone Pair Descriptor. “L.” and “R.” denote Left and Right, respectively.

MSRA, UTD-MHAD, UTK, SYSU	FLORENCE	KARD
Spine–Head (central)	Spine–Head (central)	Spine–Head (central)
Elbow R.–Wrist R.	Elbow R.–Wrist R.	Elbow R.–Wrist R.
Wrist R.–Hand R.	Shoulder R.–Elbow R.	Shoulder R.–Elbow R.
Shoulder R.–Elbow R.	Elbow L.–Wrist L.	Elbow L.–Wrist L.
Elbow L.–Wrist L.	Shoulder L.–Elbow L.	Shoulder L.–Elbow L.
Wrist L.–Hand L.	Hip R.–Knee R.	Hip R.–Knee R.
Shoulder L.–Elbow L.	Knee R.–Ankle R.	Knee R.–Foot R.
Hip R.–Knee R.	Hip L.–Knee L.	Hip L.–Knee L.
Knee R.–Ankle R.	Knee L.–Ankle L.	Knee L.–Foot L.
Ankle R.–Foot R.		
Hip L.–Knee L.		
Knee L.–Ankle L.		
Ankle L.–Foot L.		

**Table 4:** Parameters of the classifiers.

Classifier	Parameter Name	Parameter Value
DTW	Window size	5
	Metric	Euclidean
	Triplets factor	20
LDMLT	Alpha factor	5
	Number of iterations	15
	Maximum number of mixture components	5
TCK	Number of randomizations	50
	Number of iterations	20

**Table 5:** Experimental comparison of augmentation methods for three classifiers in terms of classification accuracy. The two best results for each classifier and dataset are written in bold.

Dataset/Aug. Method	None	WW	WS	SPAWNER	ARSPAWNER
DTW					
MSRA I	71.7	70.6	74.3	**74.4**	**76.1**
MSRA II	69.0	69.7	**73.1**	69.3	**71.7**
MSRA III	83.9	84.2	84.0	**86.5**	**86.5**
UTD-MHAD	86.3	86.3	83.9	**86.5**	**86.7**
UTK	81.9	80.7	**86.4**	85.4	**86.4**
FLORENCE	78.6	78.4	**81.7**	81.5	**81.8**
SYSU	69.2	67.2	70.8	**71.2**	**72.5**
KARD	89.6	**90.9**	**91.6**	88.0	89.7
LDMLT					
MSRA I	75.5	80.6	82.6	**86.2**	**86.5**
MSRA II	78.8	77.3	73.2	**80.6**	**83.2**
MSRA III	**90.2**	88.8	88.6	89.4	**89.6**
UTD-MHAD	**92.1**	90.4	84.4	**92.4**	89.2
UTK	91.5	92	91.9	**95.4**	**95.7**
FLORENCE	86.0	84.7	84.7	**88.5**	**87.4**
SYSU	68.8	61.4	64.4	**70.9**	**70.5**
KARD	95.9	96.4	94.0	**97.0**	**97.6**
TCK					
MSRA I	55.8	62.8	62.1	**65.7**	**66.5**
MSRA II	54.9	58.0	**58.5**	54.9	**58.1**
MSRA III	75.7	79.3	77.1	**81.7**	**81.4**
UTD-MHAD	**62.0**	56.6	57.7	**61.5**	60.3
UTK	92.6	**93.3**	**93.7**	93.2	**93.3**
FLORENCE	78.0	79.7	79.4	**81.6**	**80.4**
SYSU	62.7	62.8	62.3	**66.5**	**66.2**
KARD	85.5	88.0	**88.3**	**88.9**	85.2
Overall results					
Average rank	3.88	3.65	3.38	2.21	1.90
Geometric average rank	3.6	3.53	2.95	1.93	1.68
Count best	2	0	5	8	11
Average accuracy	78.2	78.3	78.7	80.7	80.9

**Table 6:** Comparison of CGAN and ARSPAWNER on the MSRA datasets. The best result for each dataset is written in bold.

Dataset	None	CGAN	ARSPAWNER
MSRA I	0.7075	0.7453	**0.8118**
MSRA II	0.6283	0.5487	**0.6994**
MSRA III	0.8125	0.6964	**0.8393**

**Table 7:** Performance of ARSPAWNER with active conditions.

Active Condition	MSRA I	MSRA II	MSRA III
Equations (2)–(3)	76.1	71.7	86.5
Equation (2)	76.1	70.4	86.5
Equation (3)	76.0	70.1	84.9
Lack of $d_{1}$ in Equations (2)–(3)	76.5	70.1	85.6
Lack of $d_{2}$ in Equations (2)–(3)	75.6	70.8	85.6

## Data Availability

In this study, publicly available datasets were analyzed. They can be found here: 1. MSRA (I, II, III)—https://sites.google.com/view/wanqingli/data-sets/msr-action3d; 2. UTD—https://personal.utdallas.edu/~kehtar/UTD-MHAD.html; 3. UTK—http://cvrc.ece.utexas.edu/KinectDatasets/HOJ3D.html; 4. FLORENCE—https://www.micc.unifi.it/resources/datasets/florence-3d-actions-dataset; 5. SYSU—http://www.isee-ai.cn/~hujianfang/ProjectJOULE.html; 6. KARD—https://data.mendeley.com/datasets/k28dtm7tr6/1; The source codes of our methods can be found here: http://vision.kia.prz.edu.pl (accessed on 13 March 2022).
